# A presumed homologue of the regulatory subunits of eIF2B functions as ribose-1,5-bisphosphate isomerase in *Pyrococcus horikoshii* OT3

**DOI:** 10.1038/s41598-018-20418-w

**Published:** 2018-01-30

**Authors:** Prerana Gogoi, Shankar Prasad Kanaujia

**Affiliations:** Department of Biosciences and Bioengineering, Indian Institute of Technology Guwahati, Guwahati, 781039 Assam India

## Abstract

The homologues of the regulatory subunits of eukaryotic translation initiation factor 2B (eIF2B) are assumed to be present in archaea. Likewise, an ORF, PH0208 in *Pyrococcus horikoshii* OT3 have been proposed to encode one of the homologues of regulatory subunits of eIF2B. However, PH0208 protein also shares sequence similarity with a functionally non-related enzyme, ribose-1,5-bisphosphate isomerase (R15Pi), involved in conversion of ribose-1,5-bisphosphate (R15P) to ribulose-1,5-bisphosphate (RuBP) in an AMP-dependent manner. Herein, we have determined the crystal structure of PH0208 protein in order to decipher its true function. Although structurally similar to the regulatory subunits of eIF2B, the ability to bind R15P and RuBP suggests that PH0208 would function as R15Pi. Additionally, this study for the first time reports the binding sites of AMP and GMP in R15Pi. The AMP binding site in PH0208 protein clarified the role of AMP in providing structural stability to R15Pi. The binding of GMP to the ‘AMP binding site’ in addition to its own binding site indicates that GMP might also execute a similar function, though with less specificity. Furthermore, we have utilized the resemblance between PH0208 and the regulatory subunits of eIF2B to propose a model for the regulatory mechanism of eIF2B in eukaryotes.

## Introduction

The resemblance of the protein translation initiation process between eukaryotes and archaea has long been postulated^1^. This notion has been well supported by the availability of homologous components, especially the translation initiation factors in both the domains of life^2–4^. With the advent of the genomic DNA sequence of hyperthermophillic archaeon, *Methanococcus jannaschii*, a considerably substantial number of homologues of eukaryotic translation initiation factors (viz. eIF1, eIF1A, eIF2, eIF4A, eIF5A, eIF5B and eIF6) have been identified^1,4–6^. The availability of these translation initiation factors in archaea reinforces the fact that archaea imitates the eukaryal mechanism of translation initiation.

This apparent understanding of the archaeal translation initiation, however, remains questionable owing to the uncertainty concerning the presence of a functional homologue of eIF2B in archaea. In eukaryotes, eIF2B functions as a guanine exchange factor (GEF) for eukaryotic translation initiation factor 2 (eIF2) and is comprised of two copies each of α, β, δ, γ and ε subunits^7^. The α, β and δ subunits form the regulatory subcomplex, while γ and ε make up the catalytic subcomplex^8,9^. The regulatory subunits of eIF2B execute its function by interacting with the α-subunit of eIF2 via the N-terminal domains^10,11^. However, the actual mechanism underlying the regulatory mechanism of eIF2B remains unspecified. Earlier studies have suggested that under stressed conditions, eIF2α is phosphorylated to form eIF2α-P which shows a higher affinity towards eIF2B. Thus, the substrate, eIF2α is converted into a competitive inhibitor (eIF2α-P) of eIF2B which ultimately results in a global downregulation of protein synthesis^12–15^.

In archaea, the homologues of only the regulatory subunits of eIF2B have been reported to be present and not the catalytic subunits^1,16^. However, the regulatory subcomplex alone would not confer full functionality to eIF2B, as the catalytic subcomplex accomplishes the crucial function of GTP exchange^17,18^. In *Pyrococcus horikoshii* OT3, a hyperthermophillic archaeon, three open reading frames (ORFs) (PH0440, PH0702 and PH0208) were postulated to encode aIF2Bα, aIF2Bβ and aIF2Bδ, respectively^19^. This assumption was merely based on sequence similarity to the α, β, and δ subunits of eIF2B and lacked convincing evidence. Later, based on an in-depth *in silico* analysis, we have demonstrated that the protein PH0208 encode an enzyme, ribose-1,5-bisphosphate isomerase (R15Pi) (EC 5.3.1.29) with all the essential amino acid residues conserved^20^. The enzyme, R15Pi was recently identified in *Thermococcus kodakarensis* (*Tk*-R15Pi) and is known to play an indispensable role during the nucleoside 5′-monophosphate (NMP) degradation pathway available only in archaea^21^. The NMP degradation pathway partially compensates for the lack of a pentose phosphate pathway in archaea and ultimately leads to the formation of 3-phosphoglyceric acid, a crucial intermediate in glycolysis and Calvin cycle^22,23^. In NMP degradation pathway, R15Pi catalyzes the conversion of ribose-1,5-bisphosphate (R15P, substrate) into ribulose-1,5-bisphosphate (RuBP, product) in an adenosine 5′-monophosphate (AMP)-dependent manner. AMP acts as an activator of R15Pi and in its absence the enzymatic activity has been found to decline dramatically. Apart from AMP, guanosine 5′-monophosphate (GMP) also elevates the activity of R15Pi, though to a lesser extent as compared to AMP. While, neither cytosine 5′-monophosphate (CMP) nor thymidine 5′-monophospahte (TMP) possesses the ability to increase the enzymatic activity of R15Pi^22^. The three-dimensional structure of R15Pi from *T. kodakarensis* has been solved, both in the presence of the substrate and the product^21^. However, due to the unavailability of an AMP/GMP-bound structure, the exact role of purine nucleotides still remains obscure.

In this study, we attempt to abolish the uncertainty regarding the (un)availability of a homologue of the regulatory subunits of eIF2B in archaea by performing a meticulous characterization of PH0208 protein. We have determined the three-dimensional structure of PH0208 protein in complex with the cognate substrate as well as product of the enzyme, R15Pi. To understand the role of purine nucleotides in structure and activity, crystal structures of PH0208 protein (wild type and mutants) in complex with only AMP, only GMP and both AMP & GMP were also deciphered. Furthermore, a comparison between the PH0208 protein and the regulatory subunits of eIF2B has been drawn to obtain an in-depth understanding of the (dis)similarities that lies between them. Finally, a model depicting the regulatory mechanism of eIF2B in eukaryotes has been proposed based on the similitude perceived between PH0208 protein and the regulatory subunits of eIF2B.

## Results

### The overall structure

**Figure 1:**
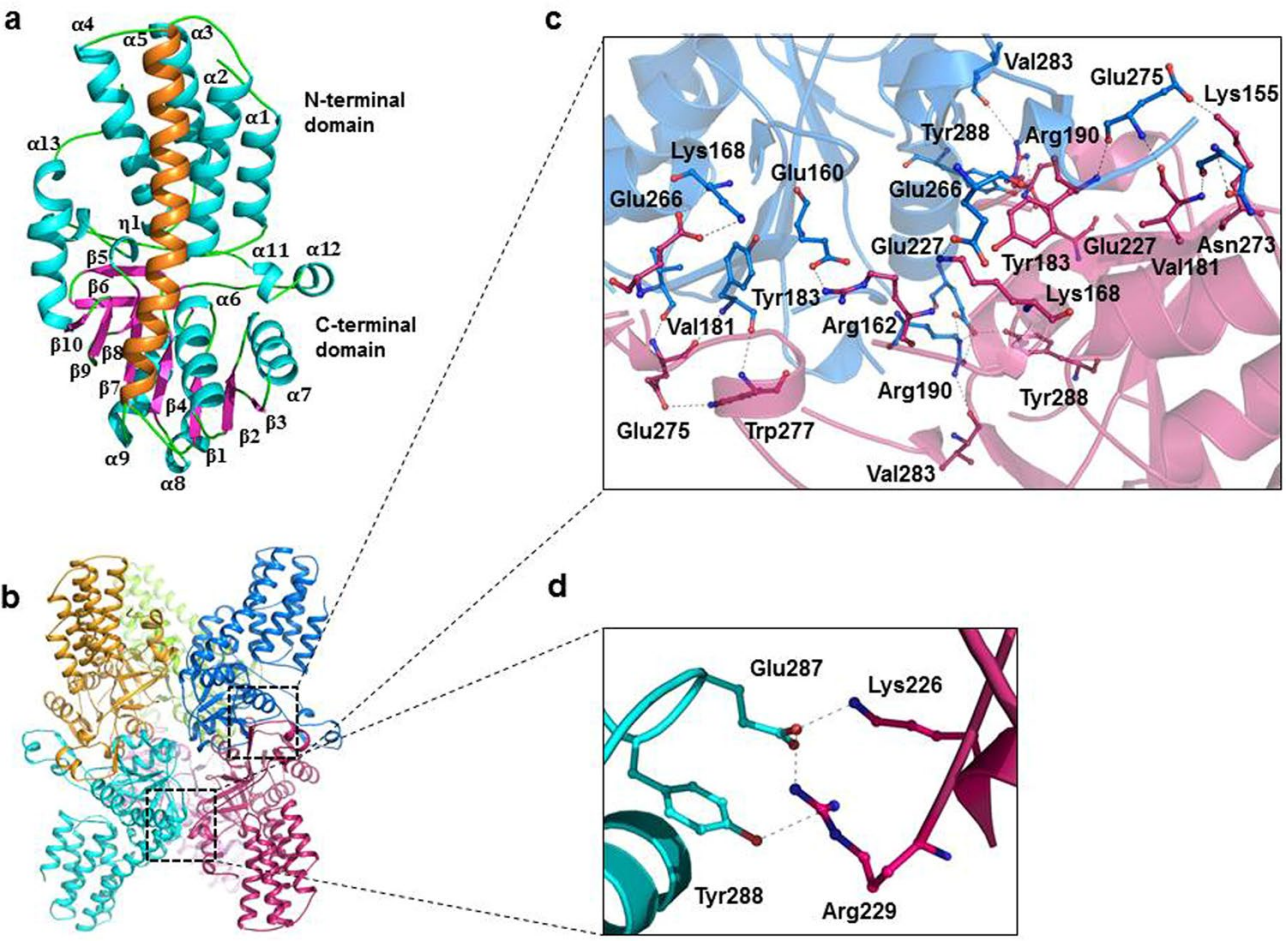
The overall structure and inter-subunit interactions (**a**) Monomer and (**b**) hexamer (chain A-F) of PH0208 protein. In the monomer, the α-helices, β-strands and loops are shown in cyan, magenta and green, respectively. The α_5_ helix connecting the N- and C-terminal domain is highlighted in orange. Each subunit is represented in different colors. Amino acid residues involved in inter-subunit interaction between (**c**) dimer and (**d**) hexamer of PH0208 protein. The amino acid residues involved in interaction are shown in ball-and-stick model.

The crystal structure of PH0208 protein was solved by molecular replacement method using the atomic coordinates of *Tk*-R15Pi (PDB id: 3VM6), which has a sequence identity of 86% with PH0208. One asymmetric unit (ASU) consists of three identical (root mean square deviation, RMSD: 0.16 Å for 321 C^α^ atoms of 324 residues) subunits of the protein. Monomer of PH0208 protein consists of an N-terminal α-helical domain (residues 1–122) and a C-terminal α-β-α sandwich domain (residues 123–324). The N-terminal domain consists of five α-helices (α_1_-α_5_) while the C-terminal domain adopts a Rossmann-like fold with eight α-helices (α_6_-α_13_), two 3_10_-helices (η_1_ & η_2_) and ten, mostly parallel β-sheets (β_1_-β_10_) (Fig. 1a). The N- and C-terminal domains are connected by the longest helix, α_5_ (residues 90–120), of the protein. Structural homology search using DALI web server^24^, reveals that PH0208 protein is closely related to proteins belonging to PF01008 family of Pfam database^25^ with RMSD ranging from 0.5–2.5 Å. This family, also known as eIF2B-related, includes the regulatory (α, β and δ) subunits of the translation initiation factor, eIF2B and methylthioribose-1-phosphate isomerase (M1Pi), an enzyme involved in methionine salvage pathway. The closest homologues of PH0208 include R15Pi from *T. kodakarensis* (PDB id: 3VM6, Z-score: 51.3), MTNA from *Bacillus subtilis* (PDB id: 2YVK, Z-score- 37.5), aIF2Bα from *P. horikoshii* (PDB id: 1VB5, Z-score: 35.6), eIF2B regulatory subunits from *Schizosaccharomyces pombe* (PDB id: 5B04, Z-score: 33–30) and *Chaetomium thermophilum* (PDB id: 5DBO, Z-score: 28.5–26.5).

The enzyme R15Pi from *T. kodakarensis* has been reported to be present as a hexamer in aqueous solution^21^. Thus, the hexameric oligomer of PH0208 was generated using the program PISA^26^. PH0208 forms hexamer with a buried surface area of ~14866 Å^2^. The interactions among the subunits of the hexamer are largely mediated by the C-terminal domain while the N-terminal domain remains completely dissociated (Fig. 1b). The interaction between amino acid residues at the C-terminal domain of two monomers lead to the formation of a dimer with a buried surface area of ~4109 Å^2^. Out of the many amino acid residues involved in dimer formation, Lys155, Arg162 and Lys168 form salt bridges with Glu275, Glu160 and Glu266 of the associating monomer, respectively (Fig. 1c). Three dimers together constitute the hexamer through interactions involving the residue Lys226 of one monomer and Glu287 of the neighboring monomer, while the residue Arg229 participates in hydrogen bonding with the residues Glu287 and Tyr288 of the adjacent monomer (Fig. 1d).

### The substrate/product of R15Pi binds at the active site pocket of PH0208 protein

**Figure 2:**
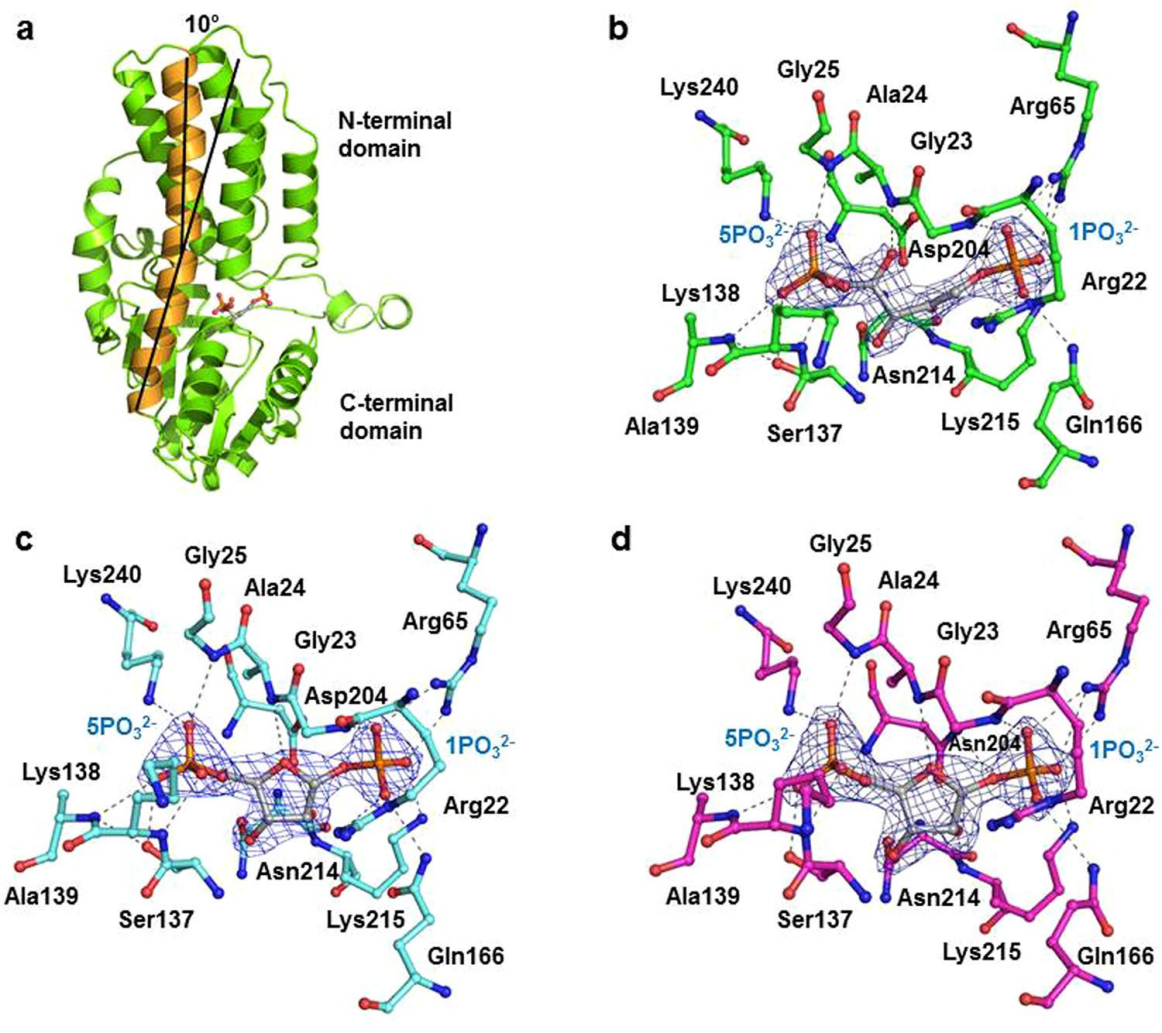
The active site pocket of PH0208 protein. (**a**) Closed conformation of PH0208 protein bound to RuBP. The bend angle (~10°) of α_5_ helix highlighted in orange signifies the closed conformation. Active site residues of (**b**) PH0208-WT protein bound to the product, RuBP, (**c**) PH0208-C135S and (**d**) PH0208-D204N mutant protein bound to the substrate, R15P. The substrate (R15P) and the product (RuBP) are shown in grey using ball-and-stick model and the phosphate groups are labeled in blue. Blue meshes indicate the *2F*_*O*_*-F*_*C*_ maps for RuBP and R15P contoured at 1.0σ. All the amino acid residues involved in interaction are labeled and shown in ball-and-stick model.

Based on the hypothesis that PH0208 might encode the enzyme R15Pi^20^, crystallization of the protein was attempted in the presence of the substrate (R15P). The active site pocket of PH0208 protein resides in the cavity formed between the N- and C-terminal domains and was found to hold the product (RuBP) indicating that the substrate was converted into product during the process of crystallization. However, unlike the homologous proteins, PH0208 remains in a closed conformation even when the product is bound to the active site. This closed conformation is reflected by the bend angle of the α_5_ helix, which is ~10° for the closed conformation (Fig. 2a).

In the active site pocket, the 1-phosphate group of RuBP forms hydrogen bond to the side chains of the residues Arg22, Arg65, Gln166, Lys215 and the backbone amide group of Gly23. The 5-phosphate group forms hydrogen bond to the side chains of the residues Ser137, Lys240 and the backbone amide group of Ala24, Gly25, Lys138 and Ala139. The side chain atoms of Asp204, Asn214 and Lys215 interact with the oxygen atoms of the ribulose sugar (Fig. 2b). The amino acid residues of PH0208 protein involved in interacting with the 1-phosphate, sugar and 5-phosphate group of RuBP are well conserved in all R15Pi enzymes. The two catalytic residues, Cys135 and Asp204 (Cys133 and Asp202 in *Tk*-R15Pi) essential for the enzymatic activity of R15Pi are also conserved in PH0208 protein^20^.

In order to verify the role of the catalytic residues in PH0208 protein, the residue Cys135 was mutated to serine. The mutation led to the formation of a catalytically inactive protein which lost the ability to convert the substrate into product and thus, held the substrate in the active site pocket (Fig. 2c). The substrate molecule (R15P) binds to the active site of PH0208-C135S in a manner similar to that of the wild type (WT) protein (Fig. 2c). The other catalytic residue, Asp204, reported to be vital for ligand binding in R15Pi enzyme was mutated to asparagine. However, quite surprisingly, a clear electron density of the substrate was still observed at the active site pocket of the PH0208-D204N mutant protein. The interactions involved in holding R15P in the active site pocket of PH0208-D204N mutant protein are equivalent to those of the PH0208-WT and PH0208-C135S proteins (Fig. 2d).

### Both the substrate (R15P) and the product (RuBP) show high binding affinity towards PH0208 protein

To determine the binding affinity of the substrate (R15P) and the product (RuBP) to the protein PH0208, isothermal titration calorimetry (ITC) experiments were performed. Since binding of the substrate to the PH0208-WT protein triggers a conformational change along with the formation of the product, the heat change measured during the ITC experiment would reflect the sum of heat generated and absorbed during each event. As a result, the binding affinity (K_d_) of PH0208 and R15P/RuBP obtained would be delusional. Therefore, binding affinity was studied using the mutant proteins, PH0208-C135S and PH0208-D204N in which case the heat change would be solely due to ligand binding.

**Figure 3:**
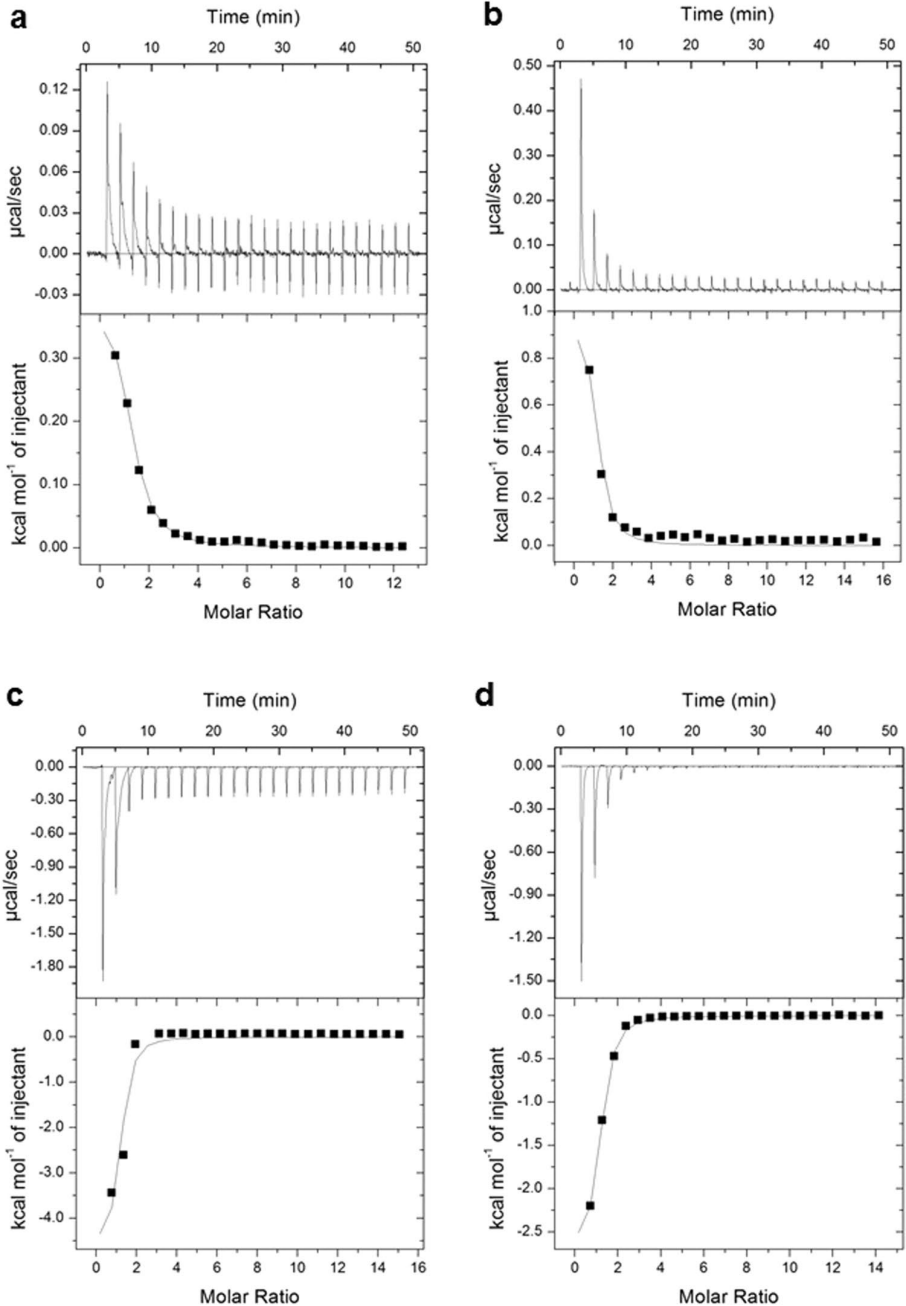
Biophysical characterization of PH0208 protein. Isothermal titration calorimetry (ITC) for the binding of (**a**) R15P to PH0208-C135S, (**b**) R15P to PH0208-D204N, (**c**) RuBP to PH0208-C135S and (**d**) RuBP to PH0208-D204N. The upper panel shows the heat change elicited upon successive injections of ligand into the protein. The lower panel shows the binding isotherm as a function of the molar ratio of ligand to protein. A theoretical curve was fitted to the integrated data using a single-site model.

The analysis shows that both PH0208-C135S and PH0208-D204N proteins bind to R15P with high affinity with equilibrium dissociation constants (K_d_) of 13.8 and 10 μM, respectively (Table 1). The raw data for both the mutants display an endothermic binding to R15P based on the positive values observed for the peak (Fig. 3a,b). Positive ∆H (enthalpy) value indicates disruption of energetically favorable interactions between the atoms. However, from the crystal structure it is evident that R15P binds to the active site pocket. Since the ∆H reflects the energy change in the entire system upon ligand binding, positive value of ∆H upon R15P binding might be contributed due to the disruption of interactions formed between the amino acid residues and water molecules in the active site pocket during the shift from an open to close conformation, which dominates the heat released upon ligand binding.

**Table 1:** Isothermal titration calorimetry (ITC) data for the binding of R15P/RuBP to PH0208-C135S, PH0208-C135A and PH0208-D204N mutant proteins.

Protein & ligand	N	∆H (kcal mol^−1^)	T∆S (kcal mol^−1^)	K_a_ (M^−1^)	K_d_ (μM)	∆G (kcal mol^−1^)
PH0208-C135S & R15P	1.17 ± 0.03	0.403 ± 0.013	7.03	7.20 × 10^4^ ± 7.13 × 10^3^	13.8	−6.6
PH0208-D204N & R15P	1(fixed)	0.981 ± 0.049	7.77	9.91 × 10^4^ ± 2.63 × 10^3^	10	−6.8
PH0208-C135S & RuBP	1 (fixed)	−4.74 ± 0.39	2.24	1.33 × 10^5^ ± 7.21 × 10^4^	7.5	−6.98
PH0208-D204N & RuBP	1.02	−2.95 ± 0.06	4.05	1.39 × 10^5^ ± 1.20 × 10^4^	7.2	−7.0
PH0208-C135A & RuBP	1 (fixed)	−8.94 ± 0.40	−4.14	3.23 × 10^3^ ± 299	309	−4.8

On the contrary, the PH0208-C135S and PH0208-D204N bind the product (RuBP) with negative ∆H value for the binding peaks indicating an exothermic reaction (Fig. 3c,d). The mutants formed strong interactions with RuBP with K_d_ values of 7.5 and 7.2 μM, respectively, which corresponds to the binding affinity of the substrate (Table 1).

### The AMP molecule binds at the interface of three protomers and provides oligomeric stability to the protein

**Figure 4:**
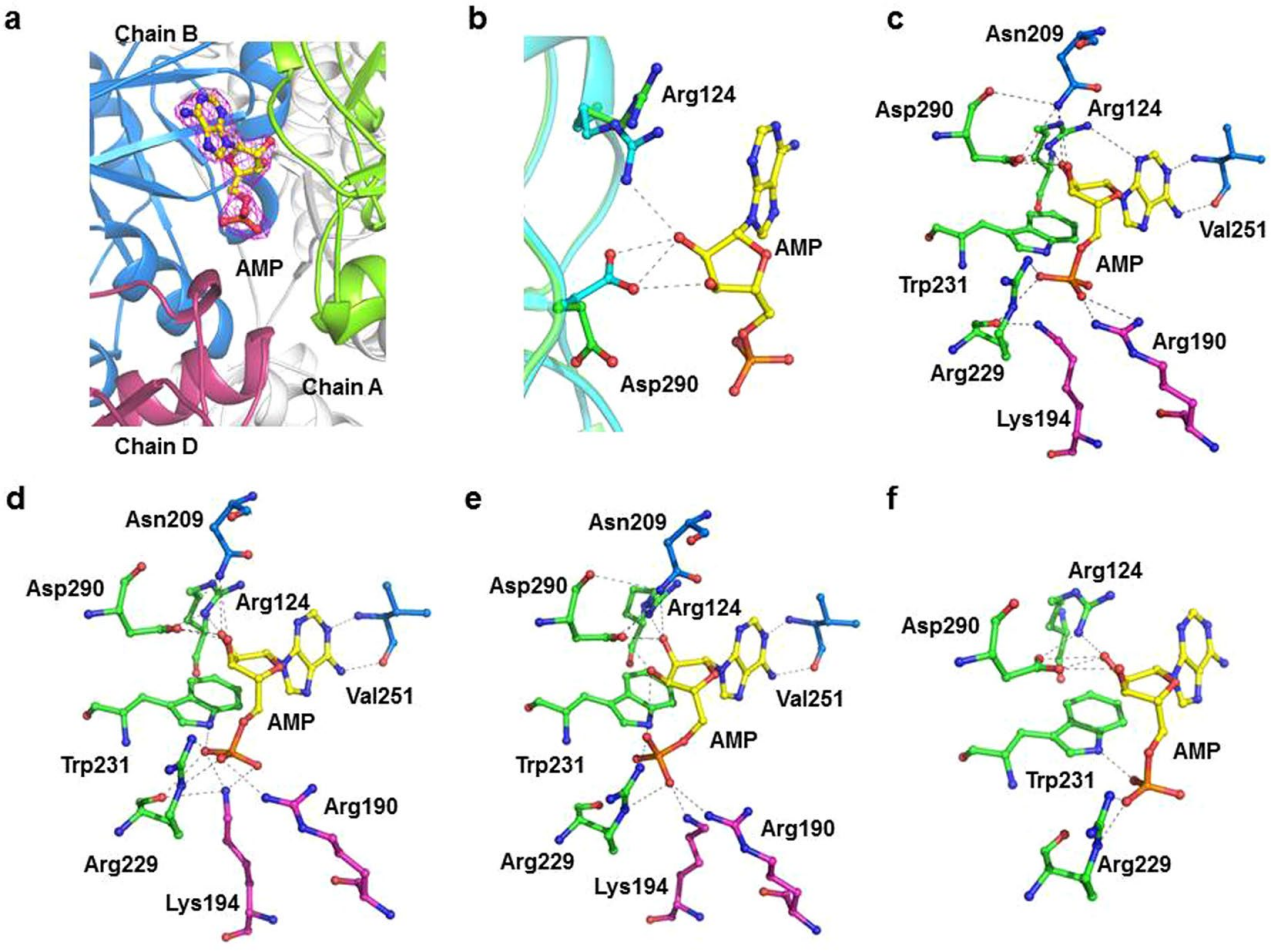
AMP molecule binds at the interface of three protomers. (**a**) The AMP molecule (shown in yellow-colored ball-and-stick model) bound at the interface of three monomers of PH0208 protein. Magenta meshes indicate the *2F*_*O*_*-F*_*C*_ map for AMP contoured at 1.0σ. Chain A, B and D of PH0208 protein interacting with AMP are shown in green, blue and magenta, respectively. (**b**) Close up view of the shift in the side chain orientation of Arg124 and Asp290 upon AMP binding. Arg124 and Asp290 of PH0208-WT and PH0208-WT•AMP complex are shown in green and cyan-colored ball-and-stick model, respectively. Close up view of AMP interacting with the amino acid residues from three different monomers of (**c**) PH0208-WT, (**d**) PH0208-C135S, (**e**) PH0208-D204N (obtained through soaking) and (**f**) PH0208-D204N (obtained through co-crystallization). The amino acid residues involved in hydrogen bond interaction are shown as ball-and-stick models in different colors for each subunit.

To identify the binding site of ribonucleotides, either co-crystallization or soaking of PH0208-WT, PH0208-C135S and PH0208-D204N proteins with AMP, GMP, CMP and TMP was performed. Complex of AMP and GMP bound to the proteins were obtained, however, no electron density for CMP or TMP was observed. The binding site of AMP lies at the interface of three monomers (chain A, B and D) of the hexameric protein (Fig. 4a). The side chains of Arg124 and Asp290 of chain A undergoes a shift towards AMP to form hydrogen bond with the O2′ and O3′ oxygen atoms of the ribose sugar (Fig. 4b). Another two residues of chain A, Arg229 and Trp231 are involved in interacting with the phosphate groups of the AMP molecule. The side chain atoms of Asn209 and the backbone atoms of Val251 of chain B forms hydrogen bond with the oxygen (O2′ and O3′) atoms of the ribose sugar and the nitrogen (N1 and N6) atom of the adenosine base ring, respectively. Along with chain A and chain B, AMP also binds to a third protomer of the hexameric protein through interactions involving the side chain atoms of Arg190 and Lys194 of chain D (Fig. 4c). Thus, one AMP molecule positions itself to hold three protomers of the hexamer. All the amino acid residues of PH0208 protein forming hydrogen bond with AMP are found to be absolutely conserved in R15Pi enzymes. Mutation of the two catalytic residues (Cys135 and Asp204) did not alter the binding behavior of AMP to the protein. In case of both the mutants, PH0208-C135S and PH0208-D204N, AMP occupies a similar position to that of PH0208-WT protein with the major interactions arising from the three subunits of the hexamer. Similar to PH0208-WT, the adenosine base ring interacts with Val251 of chain B, the oxygen atoms of ribose sugar interacts with Arg124 & Asp290 of chain A and Asn209 of chain B while the phosphate group forms hydrogen bond with Arg229 & Trp231 of chain A and Arg190 & Lys194 of chain D of the mutant proteins (Fig. 4d–f).

### GMP binds at two different sites in PH0208 protein including the ‘AMP binding site’

**Figure 5:**
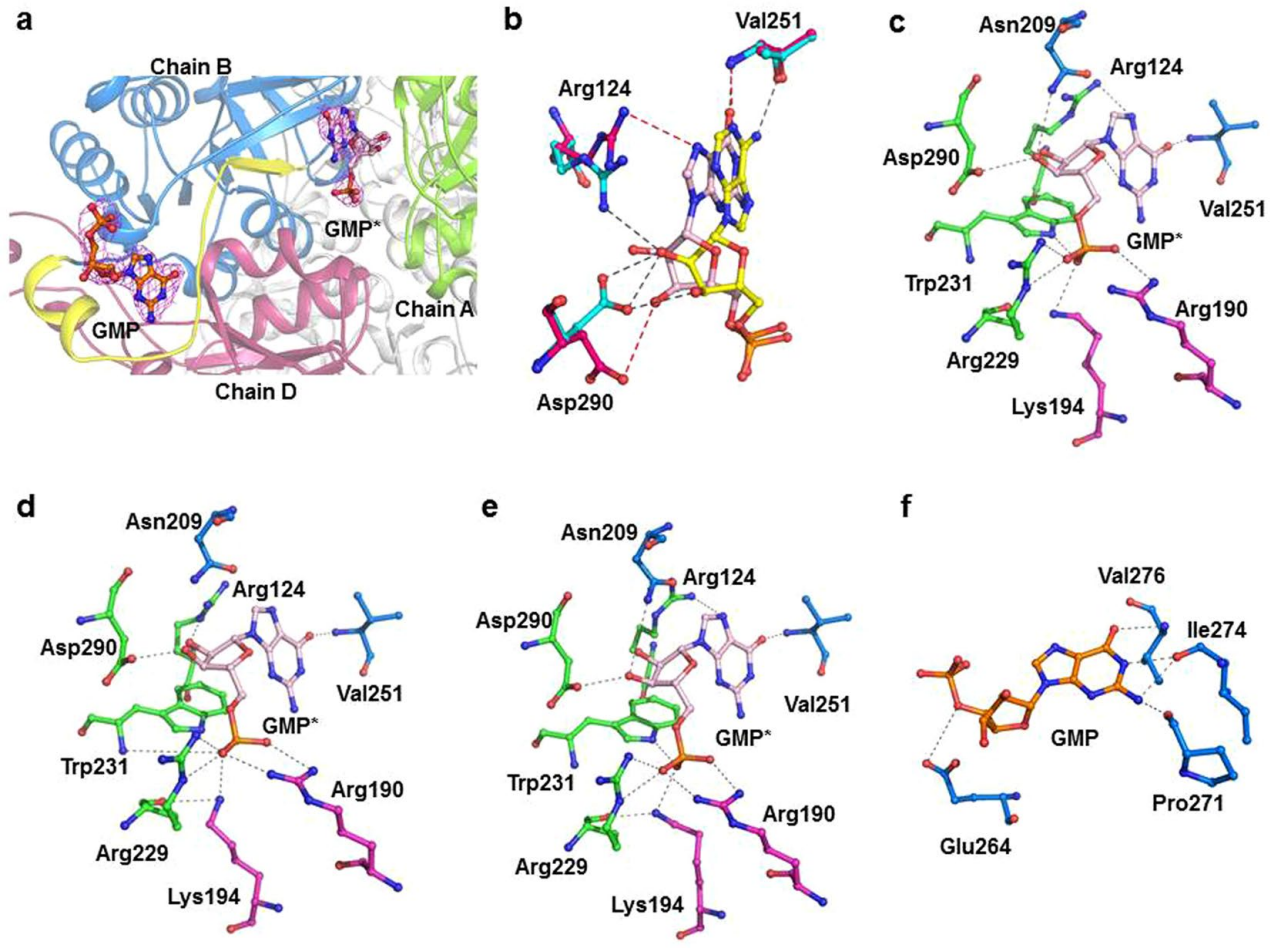
GMP binds at two different sites in PH0208 protein. (**a**) GMP bound at two different positions in PH0208 protein. GMP bound at the ‘AMP binding site’ and ‘GMP binding loop’ are shown in pink and orange-colored ball-and-stick model, respectively. Magenta meshes indicate the *2F*_*O*_*-F*_*C*_ map for GMP contoured at 1.0σ. Chain A, B and D of PH0208 protein interacting with GMP at the ‘AMP binding site’ are shown in green, blue and magenta, respectively and the ‘GMP binding loop’ between α_12_ and β_8_ is highlighted in yellow. (**b**) Close up view of the different binding modes of AMP and GMP at the ‘AMP binding site’. Arg124, Val251 and Asp290 interacting with AMP and GMP are shown as cyan and dark pink-colored ball-and-stick model, respectively. The hydrogen bonds between AMP & GMP and Arg124, Val251 and Asp290 are distinguished with grey and red dashed lines, respectively. Close up view of GMP at the ‘AMP binding site’ & the ‘GMP binding loop’ interacting with the amino acid residues from three different monomers of (**c**) PH0208-WT (**d**) PH0208-C135S and (**e**) PH0208-D204N. The amino acid residues involved in hydrogen bond interaction are shown as ball-and-stick model in different colors for each subunit. (**f**) Close up view of GMP interacting with amino acid residues of the ‘GMP binding loop’.

Interestingly, the complex structures of PH0208-WT bound with GMP reveals the binding of two GMP molecules; one GMP at the ‘AMP binding site’ and another GMP molecule at a site present in the loop region connecting α_12_ and β_8_ (hereafter referred to as ‘GMP binding loop’) (Fig. 5a). Although, GMP at the ‘AMP binding site’ is held by the same set of amino acid of PH0208-WT as in the case of AMP, there exists certain differences in the binding mode. The most pronounced difference lies in the lack of conformational change in the side chains of Arg124 and Asp290 upon GMP binding. This causes Arg124 to form hydrogen bond with N7 of the guanosine base ring instead of O2′ and Asp290 to form weak hydrogen bond with only O3′ of ribose sugar of the GMP molecule (Fig. 5b). Furthermore, unlike AMP, the amide group of the residue Val251 interacts with O6 oxygen atom of the guanosine base ring of GMP. On the other hand, similar to AMP at the ‘AMP binding site’, Arg229 & Trp231 of chain A and Arg190 & Lys194 of chain D interact with the phosphate group of GMP (Fig. 5c). Similar to PH0208-WT protein, the complex structures of PH0208-C135S and PH0208-D204N binding to GMP revealed the presence of two molecules of GMP, with one GMP at the ‘AMP binding site’ and the other at the ‘GMP binding loop’. All the interactions between the amino acid residues at the ‘AMP binding site’ of the mutant proteins and GMP molecule are equivalent to that of PH0208-WT protein (Fig. 5d,e).

At the ‘GMP binding loop’, the backbone atoms of the conserved residues Pro271, Ile274 and Val276 interacts with N2, N1 and O6 atoms of the guanosine base ring of GMP molecule, respectively. The O^ε1^ of Glu264 further interacts with O5’ while the phosphate group remain absolutely free. These interactions remain consistent in case of all the three proteins viz., PH0208-WT, PH0208-C135S and PH0208-D204N (Fig. 5f).

### AMP is preferred over GMP at the ‘AMP binding site’

**Figure 6:**
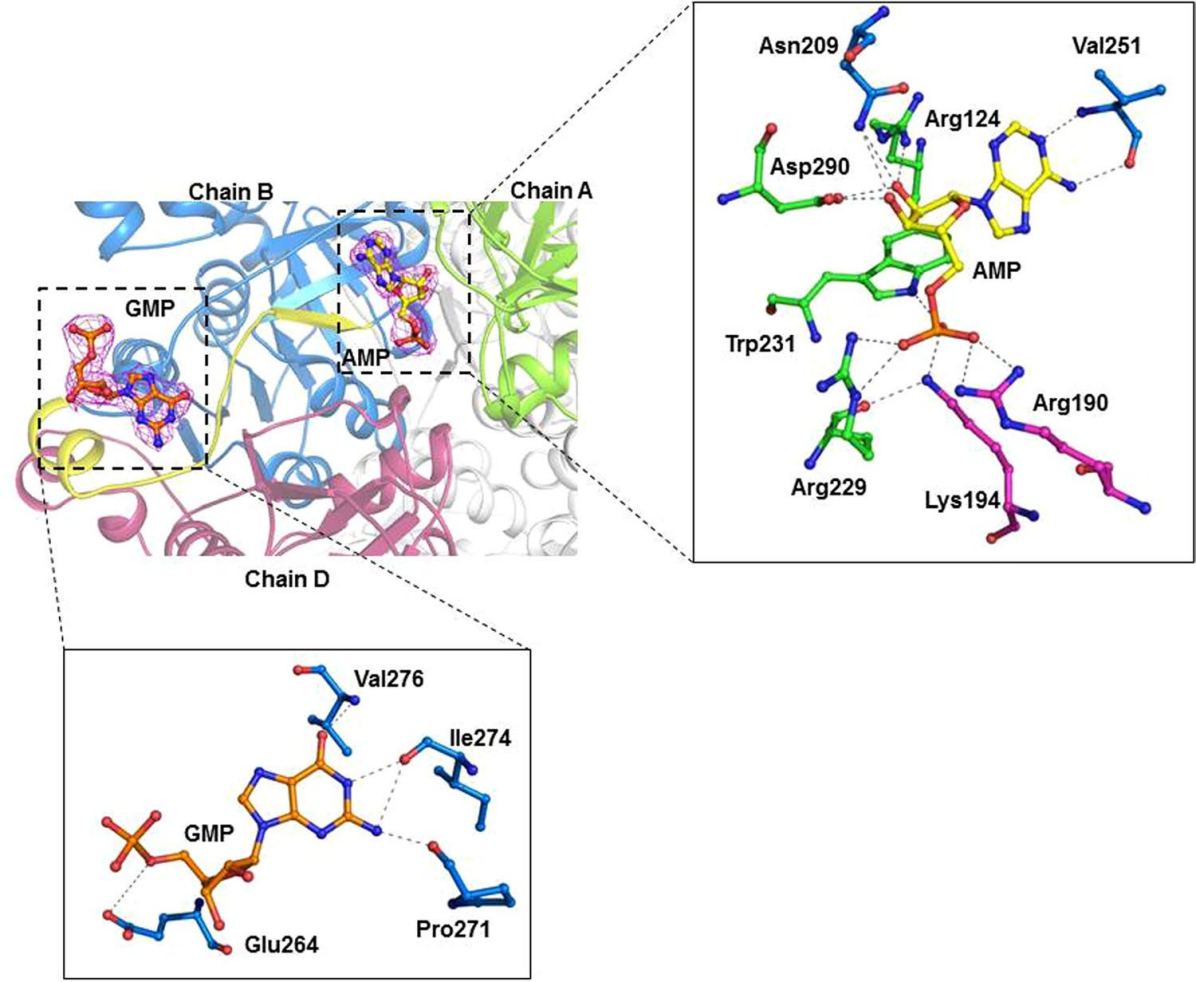
AMP is preferred over GMP at the ‘AMP binding site’. The AMP and GMP molecules bound to PH0208 protein are shown in yellow and orange-colored ball-and-stick model, respectively. Magenta meshes indicate the *2F*_*O*_*-F*_*C*_ map for AMP and GMP contoured at 1.0σ. The ‘GMP binding loop’ between α_12_ and β_8_ is highlighted in yellow. The insets show close up view of AMP (upper panel) and GMP (lower panel) interacting with amino acid residues at the ‘AMP binding site’ and ‘GMP binding loop’, respectively.

To verify the preferred molecule at the ‘AMP binding site’, PH0208-WT, PH0208-C135S and PH0208-D204N were crystallized in the presence of both AMP and GMP. In the presence of both the potential ligands, AMP outcompete GMP and binds to the ‘AMP binding site’ utilizing the similar set of amino acid while GMP binds to the ‘GMP binding loop’ (Fig. 6). During crystallization, even a higher concentration of GMP (4.5 mM) as compared to AMP (1.5 mM) could not aid GMP to occupy the ‘AMP binding site’. This further indicates a higher affinity of AMP at the ‘AMP binding site’.

### PH0208 protein is similar to eIF2Bα, albeit with crucial differences at the active site

**Figure 7:**
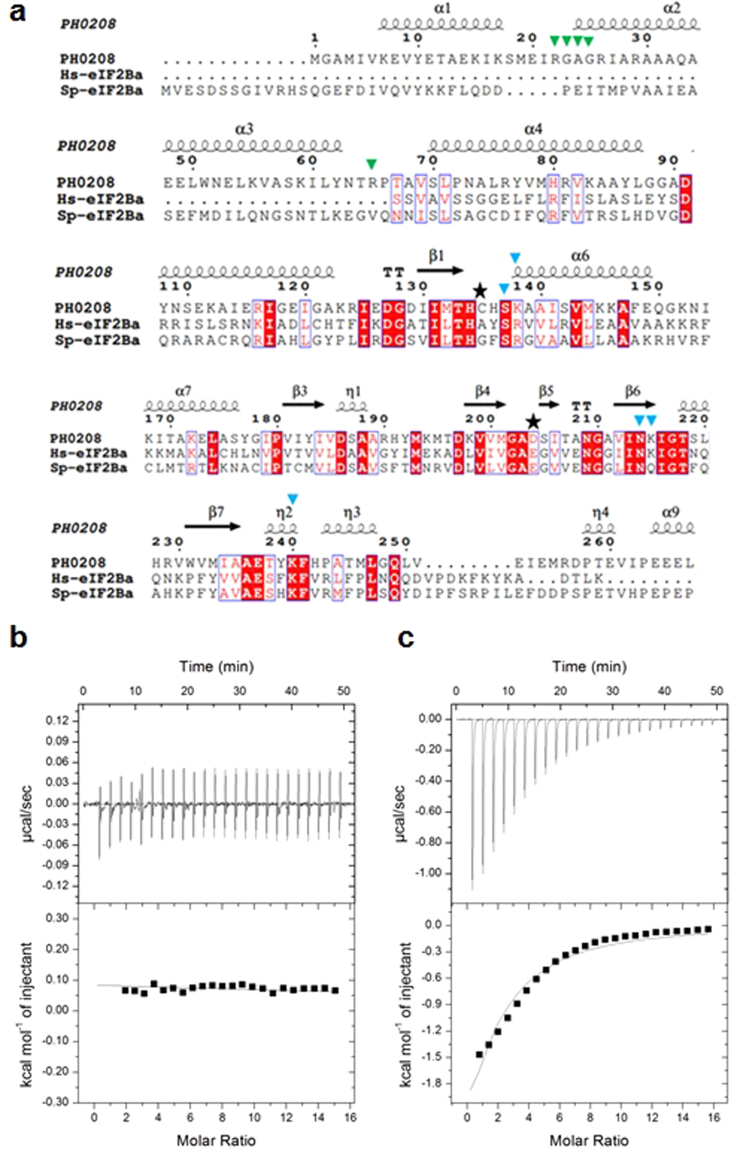
Crucial differences at the active site of PH0208 and eIF2Bα. (**a**) Multiple sequence alignment (MSA) of amino acid sequences of PH0208 (UniProt ID: O57947) and eIF2Bα from *H. sapiens* (Hs-eIF2Ba, UnitProt ID: Q14232) and *S. pombe* (Sp-eIF2Ba, UniProt ID: Q9USP0). The UniProtKB accession numbers for each protein sequences are provided in parenthesis. The residues marked with green-colored downward arrowheads are involved in interaction with the 1-phosphate group of the R15P/RuBP. The residues marked with blue-colored downward arrowheads are involved in interaction with the ribose or ribulose as well as the 5-phosphate group of R15P/RuBP. The catalytic residues are highlighted with black star. Isothermal titration calorimetry (ITC) for the binding of (**b**) R15P and (**c**) RuBP to PH0208-C135A mutant protein. The upper panel shows the heat change elicited upon successive injections of ligand into the protein. The lower panel shows the binding isotherm as a function of the molar ratio of ligand to protein. A theoretical curve was fitted to the integrated data using a single-site model.

The PH0208 protein and the regulatory subunits (α, β and δ) of eIF2B share a pronounced resemblance at the tertiary level (RMSD, eIF2Bα- 2.0 Å, eIF2Bβ- 2.44 Å and eIFBδ- 1.48 Å) with a highly similar all α-helical N-terminal and Rossmann-like fold C-terminal domain. Similar to PH0208 protein, the regulatory subunits of eIF2B assemble together by making extensive interactions via the C-terminal domain of each subunit^10,11^. Among the three regulatory subunits of eIF2B from *Homo sapiens* and *S. pombe*, PH0208 protein shares highest sequence homology with the α-subunit (Query coverage: 86 & 83%, Identity: 25 & 30%, respectively). A comparison of the active site pocket of PH0208 protein and eIF2Bα from *H. sapiens* (*Hs*eIF2Bα) & *S. pombe* (*Sp*eIF2Bα), reveals that the amino acid residues required for interacting with the 5-phosphate group and the ribose/ribulose sugar are well conserved in both *Hs*eIF2Bα and *Sp*eIF2Bα (Fig. 7a). However, the amino acid residues interacting with the 1-phosphate group of the substrate/product are absent in eIF2Bα. Out of the two catalytic residues, Cys135 is replaced by the residues alanine and glycine in *Hs*eIF2Bα and *Sp*eIF2Bα, respectively (Fig. 7a). Henceforth, Cys135 was mutated to alanine (PH0208-C135A) to understand its role in ligand (R15P/RuBP) binding. Interestingly, the mutant protein, PH0208-C135A did not show any interaction with the substrate (Fig. 7b). However, PH0208-C135A mutant protein interacted with the product albeit with a lower binding affinity (K_d_: 309 µM) as compared to the other two mutant proteins (PH0208-C135S and PH0208-D204N) (Fig. 7c, Table 1).

## Discussion

An analogy has often been drawn between the process of translation initiation in eukaryotes and archaea, primarily based on the sequence similarity of the components, especially the translation initiation factors. This belief is further strengthened by the structural similarity exhibited by most of these translation initiation factors of archaea and eukaryotes. Nevertheless, this equivalence should be drawn only after a comprehensive study of these proteins, both at the primary and tertiary structure levels. The homologues of the three regulatory subunits (α, β and δ) of eIF2B has been reported to be available in *P. horikoshii* OT3; one of them being PH0208^19^. This lacked direct evidence and required further in-depth analysis before assigning PH0208 with the function of one of the regulatory subunits of eIF2B.

The crystal structure reveals that the overall structure of PH0208 protein is similar to that of the regulatory subcomplex of eIF2B. However, the strong binding of the substrate/product of R15Pi enzyme to the active site pocket of PH0208 protein by forming interactions with all the crucial and conserved amino acid residues clearly indicates that PH0208 protein would function as R15Pi. The residues Cys135 and Asp204, known to be absolutely conserved in R15Pi enzyme and essential for the catalysis of R15P (substrate) to RuBP (product) are also conserved in PH0208 protein. Mutation of these two residues in PH0208 protein led to the inhibition of the isomerization of the substrate into product. The absence of electron density of R15P in the active site pocket of D202N (D204 in PH0208) mutant of R15Pi from *T. kodakarensis* led to the proposition that this residue might play an essential role in ligand binding^21^. Interestingly, the PH0208-D204N mutant held the substrate at the active site pocket indicating that the residue aspartate alone is not responsible for ligand binding and there might be other criteria that govern the binding of the ligand.

The ability of the AMP molecule to elevate the catalytic efficiency of R15Pi has been well documented^22^. However, the lack of information regarding the AMP binding site in R15Pi protein has limited the knowledge towards understanding the exact role of AMP during enzyme catalysis. Thus, we determined the structure of PH0208 protein in complex with AMP molecule. The positioning of the AMP at the interface of three monomers implies that AMP might play an important role in providing conformational stability to the hexameric form of PH0208 protein. The change in the side chain orientation of Arg124 and Asp290 to interact with AMP portrays as the key interactions required for this stabilization. Aono and his co-workers demonstrated the inability of compounds such as adenosine 5ʹ-triphosphate (ATP), S-adenosyl methionine (SAM) or S-adenosyl homocysteine (SAH) in increasing the catalytic efficiency of R15Pi^22^. This might be due to the steric hindrance posed by the bulky group at the C5 position of these compounds which would inhibit interaction with the residues Arg190 and Lys194. Thus, it can be inferred that Arg190 and Lys194 provide further specificity to AMP at the ‘AMP binding site’.

In the absence of AMP, binding of the GMP molecule at the ‘AMP binding site’ indicates that during AMP scarcity, GMP might occupy the same site and render similar structural stability to the protein. Although, GMP binds to the ‘AMP binding site’, the absence of the essential interactions of Arg124 and Asp290 with the ribose sugar of GMP causes the binding to be less specific. This explains the lower degree of activation of R15Pi enzyme in the presence of GMP^22^. Evidently, when both AMP and GMP were made available, AMP binds to the ‘AMP binding site’ with a higher preference than GMP, while GMP occupies the ‘GMP binding loop’ only. Binding of GMP to the ‘GMP binding loop’ involves very few interactions which predominantly includes only the guanosine base ring of the GMP molecule. Thus, it might also be possible that the binding of GMP to the ‘GMP binding loop’ is an outcome of crystallization artifact. Both, AMP and GMP bind to PH0208 protein even after the mutation of the catalytic residues (PH0208-C135S and PH0208-D204N) at the active site pocket. This implies that purine nucleotides would interact at their respective sites irrespective of whether the substrate or product is bound at the active site pocket. Interaction of the nitrogen (N1 and N6) and oxygen (O6) of adenine and guanosine base rings, respectively, with Val251 of chain B manifests the requirement of double-ring structure (purine nucleotides) at the ‘AMP binding site’. This explains the inability of pyrimidine nucleotides, CMP and TMP to bind to the ‘AMP binding site’.

Although functionally divergent, the similarity between the regulatory subcomplex of eIF2B and PH0208 protein at the structural level remains indisputable. The overall arrangement of the six subunits of PH0208 with the major interactions occurring at the C-terminal domain is comparable to the hexamer assembly of the regulatory subunits of eIF2B. The high similarity between the two proteins might originate from the fact that during the course of evolution, R15Pi underwent refunctionalization from a metabolic enzyme to form eIF2B, a translation initiation factor^10^. A detailed comparison of the active site pocket of PH0208 protein and eIF2Bα reveals the prime differences that dictate the ligand specificity to each protein. The amino acid residues of R15Pi involved in interacting with the 5-phosphate group and the ribose/ribulose sugar of R15P are also conserved in eIF2Bα. However, eIF2Bα lacks those amino acid residues required for interacting with the 1-phosphate group. Thus, it can be hypothesized that the active site pocket of eIF2Bα might accommodate a sugar-5-phosphate molecule such as ribose/ribulose-5-phosphate. Mutation of one of the catalytic residues, cysteine to alanine (PH0208-C135A) inhibits the binding of R15P to PH0208 protein. Similarly, in eIF2Bα, the cysteine residue is replaced by alanine and thus might impart further ligand specificity. From this analysis, it can be postulated that during the course of refunctionalization, eIF2Bα underwent minimum amino acid substitution at the active site pocket to change the ligand specificity.

**Figure 8:**
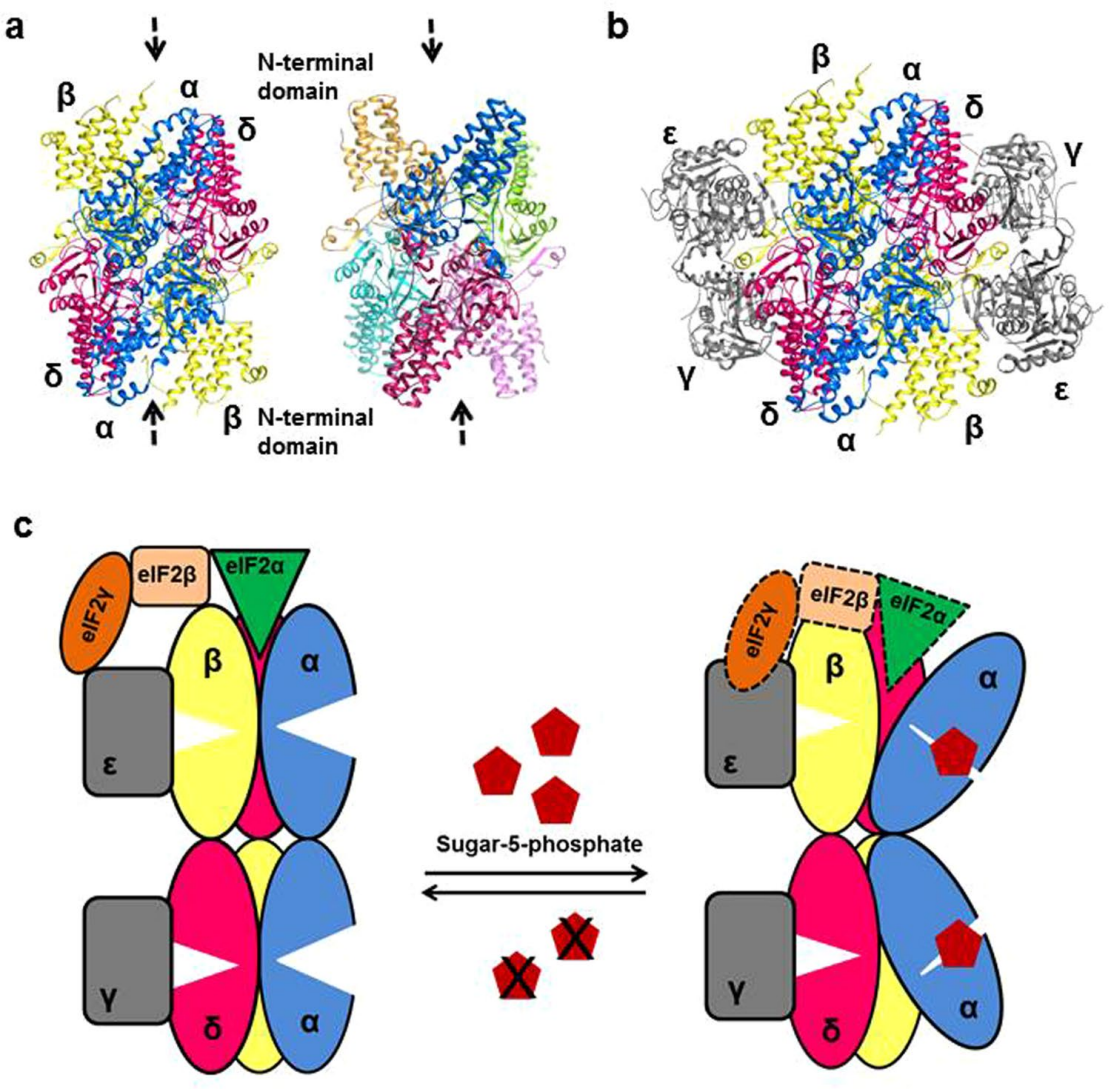
Structural similarity between PH0208 protein and the regulatory subunits of eIF2B. (**a**) Similar hexameric arrangement of the regulatory subunits of eIF2B (left) and PH0208 protein (right). The cavity formed by the N-terminal domains is shown with black dashed arrows. (**b**) Crystal structure of eIF2B from *S. pombe* (PDB id: 5B04)^11^. The regulatory subunits, α, β and δ subunits are shown in blue, yellow and magenta, respectively and the catalytic subunits, γ and ε are shown in grey. (**c**) Model for the regulatory mechanism of eIF2B. The regulatory subunits, α, β and δ are represented as blue, yellow and magenta ovals, respectively, while the catalytic subunits, γ and ε are shown as grey rounded rectangle. The α, β and γ subunits of eIF2 are represented as green arrowhead, beige rounded rectangle and orange oval, respectively. Binding of sugar-5-phosphate (brick red pentagon) to the α-subunit (blue oval) of eIF2B triggers a conformational change which creates a wider binding cavity for eIF2α (green arrowhead) at the tip of the N-terminal domain of eIF2B. The dotted lines of the eIF2 complex indicate flexible binding.

The similarity in the overall structure, significant conservedness at the active site pocket and a common evolutionary descent of R15Pi and the regulatory subunits of eIF2B indicates that the regulatory mechanism of eIF2B might utilize certain rudimentary features of R15Pi enzyme. Kuhle et al. proposed that similar to R15Pi, either all the three or fewer regulatory subunits individually undergo a close-to-open conformation, which alters the size of the cavity formed by the N-terminal domain of eIF2B regulatory subunits (Fig. 8a). The closing and opening of the cavity formed by the N-terminal domain, in turn modulates the binding affinity of eIF2α towards eIF2B^10^. Based on our study, we hypothesize that the conformational change of the α-subunit of eIF2B alone would be sufficient to modulate the binding affinity of eIF2α. Out of the three regulatory subunits, the α-subunit possesses all the essential amino acid residues required for interacting with a sugar-5-phosphate molecule. Furthermore, from the crystal structure of eIF2B^11^, it can be deduced that the binding pocket of the α-subunit only remains accessible for ligand binding whereas the binding pockets of both β- and δ- subunit are completely engulfed by the catalytic subunits (γ and ε) (Fig. 8b). Similar to PH0208 protein, the binding of a sugar-5-phosphate at the cavity formed between the N- and C-terminal domains of the α-subunit would trigger a closed conformation (Fig. 8c). The closed conformation is facilitated by the transition of the N-terminal domain which in turn would lead to an open cavity formed by the tips of the N-terminal domain of the regulatory subunits. This wider cavity would render a flexible interaction of eIF2α with eIF2B which might also aid in the process of GTP exchange by allowing more effective interaction between the catalytic subunits, eIF2γ and eIF2Bε (Fig. 8c). However, during a low energy state such as glucose deprivation, the production of sugar-5-phosphates would be less. Unavailability of sugar-5-phosphate would impede the domain movement of α-subunit of eIF2B resulting in a narrow cavity (Fig. 8c). The narrow cavity would promote a stronger interaction between eIF2α and eIF2B, thus rendering eIF2 unavailable for the subsequent rounds of translation initiation. Thus, the rate of translation initiation would be regulated in accordance to the energy state of the cell^10^.

In summary, our study demonstrates that PH0208 protein would function as a purine nucleotide dependent R15Pi enzyme and not as a homologue of the regulatory subunit of eIF2B. The outcome of this study hints towards the fact that a functional homologue of the regulatory subunits of eIF2B might not be available in archaea and similar to bacteria, archaea perhaps do not utilize a guanine exchange factor during translation initiation^27^. On the contrary, it is also likely that another unidentified ORF might accomplish the function of aIF2B in archaea. Thus, this dilemma of (un)availability of a homologue of the regulatory subunit of eIF2B impels the re-evaluation of the process of translation initiation in archaea.

## Materials and Methods

### Construction of expression plasmids

The gene encoding PH0208 was amplified using polymerase chain reaction (PCR) from the genome of *P. horikoshii* OT3 using the forward primer 5′-CGGT**CATATG**CACCATCATCATCATCATGTGGGAGCCATGATAG-3′ containing NdeI restriction site (bold) followed by a 6xHis-tag (underlined) and a reverse primer, 5′-CGA**CTCGAG**CTAATCTTCCCATGGTTCTTTATACTTTAATGCCC-3′ containing XhoI restriction site (bold). The amplified gene was inserted into pET-22b(+) excised with the same restriction enzymes. The resulting vector was further used as a template to construct the mutants, C135S, C135A and D204N using Q5 Site-Directed Mutagenesis Kit (New England Biolabs). The primers used for the construction of the mutants were as follows: C135S-F/R: 5′-ATGACACAT**TCT**CACAGCAAAGC-3′/5′-TATTATATCCCCATCCTCAATTC-3′, C135A-F/R: 5′-AATGACACAT**GCT**CACAGCAAAGCC-3′/5′- ATTATATCCCCATCCTCAATTC-3′, D204N-F/R: 5′-TATGGGAGCT**AAT**TCTATAACAGC-3′/5′-ACAACCTTATCAGTCATCTTC-3′ (point mutations are indicated in bold).

### Protein expression and purification

The recombinant plasmid constructs (PH0208-WT, PH0208-C135S, PH0208-C135A and PH0208-D204N) were transformed into Rosetta (DE3) *Escherichia coli* competent cells. The transformed *E. coli* cells were then grown at 310 K in terrific broth (TB) supplemented with 100 μg ml^−1^ ampicillin and 34 μg ml^−1^ chloramphenicol. After attaining an OD_600_ of 0.6, 3% ethanol and 1 mM isopropyl β-D-1-thiogalactopyranoside (IPTG) was added to the culture to induce soluble expression of the protein at 303 K for 12 h. The cells were harvested by centrifugation and resuspended in binding buffer containing 20 mM Tris-HCl pH 8.0, 150 mM KCl, 5 mM imidazole, 1 mM phenylmethylsulfonyl fluoride (PMSF), 3 mM β-mercaptoethanol (β-ME) and 0.2 mg ml^−1^ lysozyme. The cells were then disrupted using sonicator and the cell lysate was incubated at 363 K for 13 min to remove the thermolabile proteins deriving from the host cells. The cell lysate was then centrifuged at 12,000 rpm for 40 min at 277 K. The supernatant fraction was applied to Ni-NTA affinity column containing Ni-NTA agarose pre-equilibrated with the binding buffer without lysozyme and incubated for 2 h. The column was washed with five column-volume each of wash buffer A (20 mM Tris-HCl pH 8.0, 150 mM KCl, 10 mM imidazole, 1 mM PMSF and 3 mM β-ME) and wash buffer B (20 mM Tris-HCl pH 8.0, 150 mM KCl, 20 mM imidazole, 1 mM PMSF and 3 mM β-ME). The protein was eluted with 250 mM imidazole in 20 mM Tris-HCl pH 8.0, 150 mM KCl and 1 mM PMSF. The eluted fractions were collected and step-wise dialysis was performed against 20 mM Tris-HCl pH 8.0 and 150 mM KCl to remove imidazole. The dialyzed proteins were concentrated (PH0208-WT, PH0208-C135S, PH0208-C135A & PH0208-D204N- 9.5, 9.0, 7.0 & 7.0 mg ml^−1^, respectively) using Amicon Ultra centrifugal filter unit (Milipore) and Vivaspin turbo 15 (Sartorius) with a molecular weight cutoff of 10 kDa.

### Protein crystallization

To obtain crystals of PH0208-WT, an initial screening was performed by mixing 2 μl of protein and 2 μl of crystallization condition available in Crystal Screen, Crystal Screen 2 and PEG/Ion kits from Hampton research at 277 K and 293 K by the microbatch-under-oil technique. Further refinement of one of the conditions containing 2.0 M NaCl and 10% (w/v) PEG 6000 using hanging-drop vapor-diffusion technique in 24-well plate was performed to obtain better crystals. However, crystals of the apo form of PH0208-WT protein diffracted very poorly. To obtain diffraction quality crystals, the substrate (R15P) was added to the crystallization drop which was then equilibrated against 500 μl of 0.7 M NaCl and 3% PEG 6000. Yet, the crystals formed diffracted to a resolution of ~7 Å, only. Addition of 20–30% of 2-methyl-2,4-pentanediol (MPD) to the reservoir buffer finally resulted in better crystals which were further used for data collection. For co-crystallization of PH0208-WT, PH0208-C135S and PH0208-D204N with different ligands, the final protein concentration was kept at 9.5, 9.0 and 7.0 mg ml^−1^, respectively in 20 mM Tris-HCl pH 8.0 and 150 mM KCl. The final concentration of R15P in the crystallization drops were 8.33–9 mM while that of AMP, GMP, CMP and TMP were 1.66, 4.5 & 9, 1.66 and 9 mM, respectively. In case of ligand soaking, the concentration of AMP was kept at 5 mM. For obtaining the crystals of ligand bound PH0208-C135S and PH0208-D204N complexes, the reservoir buffer was further supplemented with 0.1–0.3% of low melting (LM) agarose. All the crystals were obtained in hanging-drop vapor-diffusion method at 20 °C within a period of 2–7 days.

### Data collection, processing and structure determination

X-ray intensity diffraction data was collected at 100 K using the home source Rigaku MicroMax-007 HF diffractometer (operated at 40 kV and 30 mA) and R-Axis IV++ imaging-plate detector available at the Central Instrument Facility (CIF) of Indian Institute of Technology Guwahati, India. The crystal to detector distance was maintained at a range of 150–200 mm. The crystals diffracted to a resolution range of 2.2 to 2.8 Å. The diffraction data were processed and scaled using the programs iMosflm and Aimless, respectively, embedded in the CCP4 package^28–30^. The intensities were converted to structure factors using the program CTRUNCATE available in CCP4 package. Details of the diffraction data statistics are given in Supplementary Tables S1–S4.

The structure of the PH0208-WT was solved by molecular replacement method using the program Phaser^31^. The three-dimensional atomic coordinates of R15Pi from *T. kodakarensis* (PDB id: 3VM6) was used as the search model (sequence identity: 86%). A total of 5% of the reflections were set aside for the calculation of R_free_^32^. The electron density for the product was observed in the active site of the protein molecule, however, water molecules were first located and added from the difference electron density maps. Subsequently, the product molecule was also modeled and refined. All the refinement were carried out using the program Refmac5 embedded in the program CCP4^30,33^. The program Coot was used to build the model^34^. A similar approach to that described above was used to solve and refine the structures of mutant proteins (PH0208-C135S and PH0208-D204N) as well as the ribonucleotide bound complexes of PH0208-WT and mutant proteins. The structure solutions were obtained using the atomic coordinates of the refined model of PH0208-WT for all the subsequent structures both in apo and holo forms.

The programs PROCHECK and MolProbity were used to check and validate the quality of the refined models^35,36^. For various analyses of the proteins and their complexes, the program PSAP was use^37^. The three-dimensional atomic coordinates and the structure factors of all the structures (PDB ids: 5YFJ, 5YFS, 5YFT, 5YFU, 5YFV, 5YFW, 5YFX, 5YG5, 5YG6, 5YG7, 5YG8, 5YG9 and 5YGA) have been deposited in the RCSB Protein Data Bank^38^. The refinement statistics and other details of the refined models are provided in Supplementary Table S1–S4.

### Isothermal titration calorimetry

ITC experiments of mutant proteins (PH0208-C135S, PH0208-C135A and PH0208-D204N) with the substrate (R15P) and the product (RuBP) molecules were measured at 25 °C using MicroCal iTC200. Before ITC experiments, the proteins were dialyzed extensively in buffer (20 mM Tris-HCl pH 8.0 and 150 mM KCl) and the equilibrated buffer was subsequently used to prepare ligand solutions to minimize the heat of dilution effects. Each titration consisted of a preliminary injection of 0.4 μl of 7.5–8.0 mM of R15P and RuBP followed by 24 injections of 1.2 μl (spacing 120 s) into the sample cell filled with protein at a final concentration of 75–80 μM. During titration, the syringe rotated at a speed of 450 rpm to facilitate adequate mixing of the reactants. Data were analyzed using MicroCal ORIGIN software and baselines were subtracted from data to obtain accurate heat exchanges^39^.

### Multiple sequence alignment

Homology searches were performed using the program BLAST^40^. The amino acid sequences of proteins used in multiple sequence alignment (MSA) were downloaded from UniProtKB database^41^. The MSA was performed using the program Clustal Omega^42^ and the aligned sequences were further decorated using the online tool ESPript^43^.

## Electronic supplementary material

Supplementary Information
