# Supplementary material for: A presumed homologue of the regulatory subunits of eIF2B functions as ribose-1,5-bisphosphate isomerase in Pyrococcus horikoshii OT3
Source: Sci Rep. 2018 Jan 30;8:1891. doi: 10.1038/s41598-018-20418-w (PMC5789824; doi:10.1038/s41598-018-20418-w)
Supplement: Supplementary file 1 — Supplementary Information [file 41598_2018_20418_MOESM1_ESM.pdf]

A presumed homologue of the regulatory subunits of eIF2B functions as ribose-1,5-bisphosphate isomerase in *Pyrococcus horikoshii* OT3

Prerana Gogoi and Shankar Prasad Kanaujia*

Department of Biosciences and Bioengineering, Indian Institute of Technology Guwahati, Guwahati – 781039, Assam, India

***Corresponding author**: Shankar Prasad Kanaujia, Associate Professor, Department of Biosciences and Bioengineering, Indian Institute of Technology Guwahati, Guwahati – 781039, Assam, India, Telephone: +91-361-2582228 (O), +91-7896024066 (M), Fax: +91-361-2582249, E-mail: spkanaujia@iitg.ernet.in and spkanaujia@gmail.com.

Supporting information

Supplementary Table S1. X-ray data and refinement statistics for PH0208-WT bound to RuBP and PH0208-C135S & PH0208-D204N mutant proteins bound to R15P. The values provided in parenthesis are for the last resolution shell.

	**PH0208-WT•RuBP**	**PH0208-C135S•R15P**	**PH0208-D204N•R15P**
Wavelength (Å)	1.5418	1.5418	1.5418
Temperature (K)	100	100	100
Space group	$P3_112$	$P3_112$	$P3_112$
Unit-cell parameter (Å, °)	*a*=*b*=98.63, *c*=257.06, *α*=*β*=90, *γ*=120	*a*=*b*=98.65, *c*=256.73, *α*=*β*=90, *γ*=120	*a*=*b*=98.81, *c*=257.08, *α*=*β*=90, *γ*=120
Resolution (Å)	71.14-2.2 (2.25-2.2)	85.58-2.3 (2.36-2.3)	85.69-2.21 (2.26-2.21)
No. of observed reflections	717401	765831	662589
No. of unique reflections	72651 (4463)	64003 (4486)	72313 (4460)
Mn(I) CC(1/2)	0.998 (0.923)	0.997 (0.942)	0.998 (0.918)
Completeness (%)	99.5 (98.7)	100 (100)	100 (99.8)
V_M (Å^3 Da^{-1})	3.30	3.30	3.32
Solvent content (%)	62.80	62.77	62.94
Mosaicity (°)	0.568	0.513	0.451
$I/\sigma(I)$	17.8 (4.5)	16.2 (5.7)	13.7 (3.9)
R_{merge}† (%)	0.088 (0.508)	0.124 (0.478)	0.117 (0.496)
R_{pim} (%)	0.043 (0.252)	0.054 (0.199)	0.056 (0.255)
R_{meas} (%)	0.098 (0.567)	0.135 (0.518)	0.139 (0.599)
Multiplicity	9.9 (9.7)	12 (13.2)	9.2 (8.1)
R_{work}/R_{free} (%)	14.97/18.97	14.39/19.82	15.12/19.34
**Protein model**			
No. of subunits in ASU	3	3	3
Protein atoms	965	966	966
Water molecules	507	615	669
RuBP	3	-	-
R15P	-	3	3
Others	9	7	10
**Deviation from ideal geometry**			
Bond length (Å)	0.018	0.018	0.02
Bond angles (°)	1.903	1.889	1.956
**Average *B*-factor (Å^2)**			
Protein atoms	36.57	29.31	30.62
Water molecules	39.69	31.96	34.20
RuBP	29.65	-	-
R15P	-	21.7	19.34

Ramachandran plot			
Favored (%)	97.91	97.81	97.91
Allowed (%)	1.77	1.77	1.77
Remaining (%)	0.31	0.42	0.31
PDB-id	5YFJ	5YFS	5YFT

† $R_{merge} = \sum_{hkl} \sum_i |I_i(hkl) - \langle I(hkl)\rangle| / \sum_{hkl}\sum_i I_i(hkl)$, where $I(hkl)$ is the intensity of reflection *hkl,* $\sum hkl$ is the sum overall reflections and $\sum i$ is the sum over *i* measurements of reflection *hkl.*

Supplementary Table S2. X-ray data and refinement statistics for PH0208-WT bound to RuBP & AMP, RuBP & GMP and RuBP, AMP & GMP. The values provided in parenthesis are for the last resolution shell.

	**PH0208-WT•RuBP•AMP**	**PH0208-WT•RuBP•GMP**	**PH0208-WT•RuBP•AMP•GMP**
Wavelength (Å)	1.5418	1.5418	1.5418
Temperature (K)	100	100	100
Space group	$P3_112$	$P3_112$	$P3_112$
Unit-cell parameter (Å, °)	a=b=98.98, c=256.61, α=β=90, γ=120	a=b=98.99, c=257.09, α=β=90, γ=120	a=b=98.59, c=256.15, α=β=90, γ=120
Resolution (Å)	85.72-2.35 (2.41-2.35)	85.73-2.75 (2.87-2.75)	85.38-2.8 (2.94-2.8)
No. of observed reflections	666605	546738	352039
No. of unique reflections	60380 (4425)	37954 (4564)	35596 (4669)
Mn(I) CC (1/2)	0.998 (0.972)	0.998 (0.962)	0.996 (0.956)
Completeness (%)	100 (100)	100 (100)	100 (100)
V_M (Å^3 Da^{-1})	3.32	3.33	3.29
Solvent content (%)	63.00	63.08	62.64
Mosaicity (°)	0.55	0.518	0.55
$I/\sigma(I)$	14.4 (3.7)	22.1 (5.9)	13.6 (4.9)
R_{merge}† (%)	0.109 (0.559)	0.113 (0.498)	0.138 (0.480)
R_{pim} (%)	0.051 (0.257)	0.044 (0.195)	0.067 (0.234)
R_{meas} (%)	0.121 (0.616)	0.122 (0.535)	0.154 (0.535)
Multiplicity	11.0 (11.3)	14.4 (14.8)	9.9 (9.9)
R_{work}/R_{free} (%)	18.01/24.78	16.24/23.22	15.52/22.66
**Protein model**			
No. of subunits in ASU	3	3	3
Protein atoms	966	971	968
Water molecules	321	193	241
RuBP	3	3	3
AMP	3	-	3
GMP	-	2	2
GMP*	-	3	-
Others	4	6	4

**Deviation from ideal geometry**			
Bond length (Å)	0.015	0.013	0.013
Bond angles (°)	1.845	1.679	1.750
**Average *B*-factor ($Å^2$)**			
Protein atoms	48.32	43.24	41.19
Water molecules	42.10	31.28	30.53
RuBP	37.96	32.61	33.39
AMP	43.57	-	39.55
GMP	-	102.9	89.01
GMP*	-	102.35	-
**Ramachandran plot**			
Favored (%)	96.66	96.35	96.98
Allowed (%)	2.71	3.13	2.4
Remaining (%)	0.63	0.52	0.63
PDB-id	5YFU	5YG5	5YG8

† $R_{merge} = \sum_{hkl} \sum_i |I_i(hkl) - \langle I(hkl)\rangle| / \sum_{hkl}\sum_i I_i(hkl)$, where $I(hkl)$ is the intensity of reflection *hkl,* $\sum hkl$ is the sum overall reflections and $\sum i$ is the sum over *i* measurements of reflection *hkl.*
* GMP bound at the 'AMP binding site'.

Supplementary Table S3. X-ray data and refinement statistics for PH0208-C135S mutant protein bound to R15P & AMP, R15P & GMP and R15P, AMP & GMP. The values provided in parenthesis are for the last resolution shell.

	**PH0208-C135S•R15P•AMP**	**PH0208-C135S•R15P•GMP**	**PH0208-C135S•R15P•AMP•GMP**
Wavelength (Å)	1.5418	1.5418	1.5418
Temperature (K)	100	100	100
Space group	$P3_112$	$P3_112$	$P3_112$
Unit-cell parameter (Å, °)	a=b=98.14, c=255.68, α=β=90, γ=120	a=b=98.57, c=256.62, α=β=90, γ=120	a=b=98.84, c=256.28, α=β=90, γ=120
Resolution (Å)	85.23-2.75 (2.87-2.75)	85.54-2.35 (2.41-2.35)	85.60-2.8 (2.94-2.80)
No. of observed reflections	292248	467995	417409
No. of unique reflections	37157 (4510)	59895 (4593)	35799 (4709)
Mn(I) CC (1/2)	0.995 (0.910)	0.995 (0.907)	0.997 (0.943)
Completeness (%)	100 (100)	100 (100)	100 (100)
V_M ($Å^3$ Da^{-1})	3.25	3.30	3.31
Solvent content (%)	62.23	62.69	62.85
Mosaicity (°)	0.55	0.5	0.524
$I/\sigma(I)$	12.0 (3.9)	11.2 (3.9)	15.6 (5.1)
R_{merge}† (%)	0.144 (0.502)	0.133 (0.497)	0.142 (0.509)

R_{pim} (%)	0.081 (0.285)	0.076 (0.287)	0.063 (0.220)
R_{meas} (%)	0.166 (0.578)	0.154 (0.574)	0.155 (0.555)
Multiplicity	7.9 (7.9)	7.8 (7.7)	11.7 (12.3)
R_{work}/R_{free} (%)	15.18/22.40	15.46/20.84	15.57/22.73
**Protein model**			
No. of subunits in ASU	3	3	3
Protein atoms	966	971	968
Water molecules	254	499	210
R15P	3	3	3
AMP	3	-	3
GMP	-	2	2
GMP*	-	3	-
Others	4	4	4
**Deviation from ideal geometry**			
Bond length (Å)	0.013	0.017	0.013
Bond angles (°)	1.744	1.840	1.747
**Average *B*-factor (Å^2)**			
Protein atoms	36.60	32.34	37.74
Water molecules	27.77	32.96	28.68
R15P	31.99	26.26	35.17
AMP	34.96	-	44.32
GMP	-	78.47	117.78
GMP*	-	105.49	-
**Ramachandran plot**			
Favored (%)	97.5	97.91	96.87
Allowed (%)	1.98	1.67	2.5
Remaining (%)	0.52	0.42	0.63
PDB-id	5YFV	5YG6	5YG9

† $R_{merge} = \sum_{hkl} \sum_i |I_i(hkl) - \langle I(hkl) \rangle| / \sum_{hkl} \sum_i I_i(hkl)$, where $I(hkl)$ is the intensity of reflection *hkl*, $\sum hkl$ is the sum overall reflections and $\sum i$ is the sum over *i* measurements of reflection *hkl*.
* GMP bound at the 'AMP binding site'.

Supplementary Table S4. X-ray data and refinement statistics for PH0208-D204N mutant protein bound to R15P & AMP, R15P & GMP and R15P, AMP & GMP. The values provided in parenthesis are for the last resolution shell.

	**PH0208-D204N•R15P•AMP (Co-crystal)**	**PH0208-D204N•R15P•AMP (Soaking)**	**PH0208-D204N•R15P•GMP**	**PH0208-D204N•R15P•AMP•GMP**
Wavelength (Å)	1.5418	1.5418	1.5418	1.5418
Temperature (K)	100	100	100	100
Space group	*P*321	$P3_112$	$P3_112$	$P3_112$

Unit-cell parameter (Å, °)	a=b=81.13, c=100.18, α=β=90, γ=120	a=b=98.7, c=256.55, α=β=90, γ=120	a=b=99.160, c=257.04, α=β=90, γ=120	a=b=98.8, c=256.43, α=β=90, γ=120
Resolution (Å)	70.26-2.7 (2.83-2.7)	85.52-2.35 (2.41-2.35)	85.87-2.5 (2.58-2.5)	85.56-2.45 (2.53-2.45)
No. of observed reflections	112204	626998	409894	671257
No. of unique reflections	10900 (1408)	60020 (4384)	46864 (4020)	53172 (4603)
Mn(I) CC (1/2)	0.998 (0.912)	0.998 (0.954)	0.999 (0.927)	0.999 (0.967)
Completeness (%)	100 (100)	100 (100)	92.7 (87.9)	100 (100)
V_M (Å^3 Da^{-1})	2.61	3.30	3.34	3.31
Solvent content (%)	52.98	62.78	63.20	62.84
Mosaicity (°)	0.764	0.683	0.567	0.527
$I/\sigma(I)$	23.0 (4.4)	16.3 (5.1)	17.6 (3.7)	19.6 (5.2)
R_{merge}† (%)	0.085 (0.513)	0.105 (0.461)	0.081 (0.504)	0.105 (0.552)
R_{pim} (%)	0.040 (0.252)	0.049 (0.222)	0.039 (0.233)	0.045 (0.212)
R_{meas} (%)	0.094 (0.572)	0.116 (0.512)	0.091 (0.559)	0.114 (0.592)
Multiplicity	10.3 (9.8)	10.4 (10.3)	8.7 (9.1)	12.6 (15.1)
R_{work}/R_{free} (%)	17.65/26.44	15.58/20.27	17.34/22.61	16.75/22.53
**Protein model**				
No. of subunits in ASU	1	3	3	3
Protein atoms	321	966	971	968
Water molecules	45	464	211	301
R15P	1	3	3	3
AMP	1	3	-	3
GMP	-	-	2	2
GMP*	-	-	3	-
Others	-	4	4	4
**Deviation from ideal geometry**				
Bond length (Å)	0.011	0.017	0.015	0.016
Bond angles (°)	1.651	1.900	1.799	1.822
**Average *B*-factor (Å^2)**				
Protein atoms	49.08	35.28	48.65	41.90
Water molecules	32.41	35.09	39.06	38.33
R15P	34.06	23.35	34.31	29.15
AMP	40.87	81.07	-	46.83
GMP	-	-	99.72	124.25
GMP*	-	-	86.60	-
**Ramachandran plot**				
Favored (%)	96.24	98.01	96.87	96.77
Allowed (%)	3.13	1.57	2.29	2.61
Remaining (%)	0.63	0.42	0.83	0.63
PDB-id	5YFX	5YFW	5YG7	5YGA

† $R_{merge} = \sum_{hkl} \sum_i |I_i(hkl) - \langle I(hkl) \rangle| / \sum_{hkl} \sum_i I_i(hkl)$, where $I(hkl)$ is the intensity of reflection *hkl,* $\sum hkl$ is the sum overall reflections and $\sum i$ is the sum over *i* measurements of reflection *hkl*.
* GMP bound at the 'AMP binding site'.
